# Supplementary material for: How childhood psychological abuse affects adolescent cyberbullying: The chain mediating role of self-efficacy and psychological resilience
Source: PLoS One. 2024 Sep 9;19(9):e0309959. doi: 10.1371/journal.pone.0309959 (PMC11383216; doi:10.1371/journal.pone.0309959)
Supplement: Attachment — Submitted filename: Review of Manuscript PONE.docx [file pone.0309959.s002.docx]

**Review of Manuscript PONE-D-24-04494**

Dear Authors,

Firstly, I would like to congratulate you on your significant contribution to the literature through this insightful article. The topic of understanding the underlying mechanisms linking childhood psychological abuse to adolescent cyberbullying is indeed crucial. Your work sheds light on potential pathways for targeted interventions and support programs aimed at promoting the well-being of adolescents facing early adversity.

**Introduction:**

While the introduction provides a clear argument about the study context and literature gaps, it can be further enhanced by addressing the following key points:

**Positioning the Study:**

The introduction should position the study within the broader context of existing literature. This involves not only identifying the gaps but also explaining why it is necessary to explore the specific objectives of this study. Highlight the importance of the study by discussing the practical and theoretical implications briefly in the introduction section. Explain how this research can contribute to the existing body of knowledge and why it is critical to understand the mechanisms linking childhood psychological abuse to cyberbullying.

**Literature Gaps:**

Provide a more in-depth discussion on the specific gaps in the literature that this article aims to address. Detail how your research will close some of these gaps, contributing to the field in a novel way.

**Research Questions (Objectives) and Contributions and Beneficiaries:**

Clearly state the research questions (RQs) and outline the principal contributions of the study. This helps in setting the expectations for the readers and provides a roadmap for the rest of the article.

Discuss the objectives of the study explicitly and identify the primary beneficiaries. For instance, mention how authorities, parents, educators, and policymakers can benefit from the findings of this research.

**Article Structure:**

Include a brief overview of the article’s structure at the end of the introduction. This should give the readers an idea of what to expect in the subsequent sections, making it easier for them to follow the narrative.

*In summary, the introduction should provide a comprehensive snapshot of the entire article. It should encapsulate the study’s significance, objectives, research questions, literature gaps, and potential beneficiaries. An effective introduction captures the reader's attention and compels them to read further, providing them with a clear understanding of what the article entails.*

**Theoretical Background and Hypothesis Development:**

To enhance the clarity and depth of the theoretical framework, I suggest renaming this section to "Theoretical Background and Hypothesis Development." This section should comprehensively cover the approaches and theories relevant to the article's context, along with a precise justification for the selection of the studied variables and their explicit definitions. Below are specific recommendations for improving this section:

**Discussion of the Theory and its importance:**

Begin with a thorough discussion of the chosen theory, highlighting its significance in enhancing our understanding of the topic. Explain why this particular theory is more suitable compared to other theories in the context of this study. This will provide readers with a strong rationale for the theoretical foundation of your research.

Elaborate on the importance of the Social Cognitive Theory (SCT) in understanding cyberbullying behaviors. Discuss its relevance and how it helps in explaining the mechanisms linking childhood psychological abuse to cyberbullying through self-efficacy and psychological resilience.

**In the following points, researchers have written some of the parts I have mentioned in my comments, so they should read my comments and add the missing parts only:**

**Justification of Variable Selection:**

Provide a more precise justification for selecting the studied variables, such as childhood psychological abuse, self-efficacy, psychological resilience, and cyberbullying. Explicitly define each variable and explain its relevance to the study. This helps in establishing a clear connection between the theory and the research variables.

**Definition and Impact of Childhood Psychological Abuse:**

Enhance the discussion on childhood psychological abuse by incorporating insights from psychology literature. Explain how childhood psychological abuse influences psychological development and brain function. *This can include references to studies on the neurological and psychological impacts of such abuse, providing a more rigorous and enriched discussion.*

**Linking Theory to Hypothesis Development:**

After establishing the theoretical foundation and defining the variables, proceed with discussing the relationships that lead to hypothesis development. Ensure that each hypothesis is clearly derived from the theoretical framework and supported by relevant literature. This logical flow will strengthen the argumentation and coherence of your hypothesis development.

By restructuring and enriching this section, you will provide a more robust theoretical foundation for your study. It will also make the rationale for your hypotheses clearer and more compelling, thus enhancing the overall quality of your manuscript.

**Methodology:**

The methodology section is crucial for validating the robustness and reliability of the study. Below are my suggestions for enhancing this section:

**Justification of Participant Number:**

Provide a detailed justification for the total number of participants. Explain how the number of 891 participants was determined. This should include considerations such as the study’s objectives, the expected effect size, and the desired power of the study.

**Sampling Design:**

Elaborate on the sampling design used in the study. Describe the sampling method (e.g., random sampling, stratified sampling) and justify why this method was chosen. Include information about how participants were selected from the four public secondary schools in Jiangsu Province and how this sampling design helps achieve a representative sample of the population.

**Sample Size Determination:**

Discuss the methods used to determine the sample size before conducting the study. This could include power analysis or other statistical methods to ensure that the sample size is adequate for detecting the expected effects. Provide details on the calculations or criteria used to arrive at the required sample size.

**Data Analysis Process:**

Clearly describe the data analysis process and procedures used in the study without presenting the results in this section. Explain the steps taken for data cleaning, handling missing data, and the statistical techniques used for analyzing the data (e.g., descriptive statistics, reliability testing, Pearson correlation analysis, structural equation modeling). Discuss the rationale for choosing these techniques and how they align with the study’s objectives.

**Relocation of Results:**

Move the results currently presented in the "2.3 Questionnaire Design" section to the Results section. This ensures a clear distinction between the methodology and the findings of the study. The methodology should focus on how the data was collected and analyzed, while the Results section should present the outcomes of these analyses.

*By incorporating these elements, the methodology section will provide a clearer, more comprehensive explanation of the study’s design and execution. This will help in establishing the credibility and reliability of the research findings.*

**Results:**

Before delving into the assessment of the structural equation model, it is essential to discuss the assessment of the measurement model. This involves evaluating the model fit, factor loadings, reliability, and validity of the constructs used in the study.

**Assessment of Measurement Model:**

**Model Fit:**

Report the fit indices for the measurement model, including chi-square (χ²), degrees of freedom (df), chi-square to degrees of freedom ratio (χ²/df), Root Mean Square Error of Approximation (RMSEA), Tucker-Lewis Index (TLI), Comparative Fit Index (CFI), Normed Fit Index (NFI), Goodness of Fit Index (GFI), and Adjusted Goodness of Fit Index (AGFI). These indices provide an overall assessment of how well the model fits the data.

**Factor Loadings:**

Present the factor loadings for each item. Factor loadings should be significant and ideally above 0.50, indicating that the items are good indicators of their respective constructs.

**Reliability:**

Cronbach's α: Report the Cronbach's alpha values for each construct to assess internal consistency. Values above 0.70 are generally considered acceptable.

**Composite Reliability (CR):** Provide the composite reliability values for each construct. CR values above 0.70 indicate good reliability.

**MaxR(H):** Discuss the MaxR(H) values, which provide an additional measure of construct reliability.

**Validity:**

Average Variance Extracted (AVE): Report the AVE values for each construct. AVE values above 0.50 suggest that the construct explains more than half of the variance of its indicators.

**Discriminant Validity:** Ensure discriminant validity by comparing the square root of the AVE for each construct with the inter-construct correlations. The square root of the AVE should be greater than the correlations with other constructs, indicating that the constructs are distinct from each other.

By providing a thorough assessment of the measurement model, the study can demonstrate the reliability and validity of the constructs used in the analysis. This foundational step ensures that the subsequent assessment of the structural equation model is based on a solid measurement framework.

**Assessment of Structural Equation Modeling:**

Proceed with the assessment of the structural equation model. This includes testing the hypothesized relationships between childhood psychological abuse, self-efficacy, psychological resilience, and cyberbullying.

Present the path coefficients, significance levels, and fit indices for the structural model.

Discuss the indirect, and total effects as hypothesized in the study. Use bootstrap analysis to confirm the significance of indirect effects.

By structuring the results section in this manner, the study provides a clear and comprehensive evaluation of both the measurement and structural models, ensuring the robustness and reliability of the findings.

**Findings and Discussion:**

The findings and discussion sections of the manuscript are sound. However, there should be a more detailed discussion on the theoretical contributions and how this research enhances existing knowledge. Additionally, the limitations section should be moved to the end, just before the conclusion. Furthermore, the article would benefit from a concluding remarks section that effectively summarizes the key points of the study.
